# Measuring movement into residential care institutions in Haiti after Hurricane Matthew: A pilot study

**DOI:** 10.1371/journal.pone.0195515

**Published:** 2018-04-16

**Authors:** Beth L. Rubenstein, Matthew MacFarlane, Celina Jensen, Lindsay Stark

**Affiliations:** 1 Department of Population and Family Health, Columbia University Mailman School of Public Health, New York, NY, United States of America; 2 Department of Epidemiology, Columbia University Mailman School of Public Health, New York, NY, United States of America; 3 Save the Children UK, London, United Kingdom; UNICEF, UNITED STATES

## Abstract

**Background:**

Governments have an ethical imperative to safeguard children in residential care institutions at all times, including in the aftermath of an emergency. Yet, a lack of accurate data about how the magnitude and characteristics of this population may change due to an emergency impedes leaders’ ability to formulate responsive policies and services, mobilize resources and foster accountability. The purpose of this study was therefore to determine the feasibility of evaluating movement of children into residential care following an emergency.

**Methods:**

The pilot study took place in Les Cayes commune in the Sud Department of Haiti in April 2017. Six months prior to the pilot, the area was severely affected by Hurricane Matthew, with widespread devastation to property, livestock and livelihoods. Using a two-stage process, the team created a comprehensive list of residential care institutions in Les Cayes. At each facility, the data collectors attempted to administer four separate tools: a group count tool, a record review tool, interviews with staff, and interviews with children 10 years of age and older.

**Results:**

Out of 27 known institutions in Les Cayes, 22 institutions consented to participate in the research. Within these 22 institutions, the prevalence of new arrivals to residential care since Hurricane Matthew varied significantly across the four tools, ranging from 0.69% according to the aggregated child interviews to 20.96% according to the aggregated staff interviews. Record availability and quality was very poor and child participation was difficult to arrange due to travel and scheduling constraints.

**Interpretation:**

Robust measurement of new arrivals to residential care institutions was not feasible in Haiti following Hurricane Matthew. Moreover, many of the challenges encountered are likely to be encountered in humanitarian emergencies in other settings. Therefore, the research team does not recommend scale-up of these methods in most humanitarian settings. Alternative approaches that incorporate household survey methods to ascertain movement into residential care based on reports from caregivers may be more realistic in places with poor pre-existing governance systems and weak registries and records for residential care institutions.

## Introduction

Children living in residential care institutions are vulnerable to multiple risks to their health and development. Compared with children in family care, children placed into residential care institutions at young ages have poorer growth trajectories and lower social, emotional and cognitive functioning [1]. These adverse outcomes were documented in several well-designed research studies from Russia and Romania [2–5]. Risks for children who are placed into residential care at school age and older are less well-understood, but may include physical and sexual abuse, trafficking and economic exploitation as well as negative social and emotional effects [6–9].

Despite the risks associated with residential care institutions, they continue to proliferate, especially in low- and middle-income countries. For example, a recent study across 24 sites in Cambodia estimated that nearly 1 out of every 100 children in Cambodia is living in residential care [10]. Reasons for the proliferation of residential care institutions are multi-faceted, but include poverty and lack of educational opportunities and social services at the community level [10–13]. Some institutions are also motivated by profit, which can lead to recruitment practices whereby parents are goaded into relinquishing their children [14]. In addition, residential care institutions serve children whose parents have died and who lack other options for care, but in most settings, true orphans constitute a small minority of the total population of children in residential care [11–13].

The occurrence of humanitarian emergencies can exacerbate all of these drivers of residential care. For example, emergencies, including conflict and natural disasters, can negatively impact economic livelihoods, particularly among rural populations who rely on livestock and agriculture. Access to school and social services may also be inhibited or interrupted due to destruction of infrastructure and/or security concerns. When layered on top of baseline poverty and food insecurity, these emergency-related challenges may lead to spikes in movement of children into residential care institutions [13, 15].

Governments have an ethical imperative to safeguard children in residential care institutions at all times, including in the aftermath of an emergency. Yet, a lack of accurate data about how the magnitude and characteristics of this population may change due to an emergency impedes leaders’ ability to formulate responsive policies and services, mobilize resources and foster accountability. Recognizing this gap in knowledge, an interagency Advisory Panel for the ‘Measuring Separation in Emergencies’ initiative recommended the need to develop and pilot methods to measure changes in the population of children in residential care institutions following an emergency. This recommendation was in addition to ongoing work overseen the Advisory Panel to develop two other methods to measure children separated from their caregivers in emergencies. The first method involved a mobile phone-based community surveillance approach and the second method relied on a household survey [15, 16]. However, neither of these methods were specifically designed to capture a complete picture of children living in residential care institutions.

The purpose of this study was therefore to evaluate the feasibility of a third method focused specifically on the measurement of changes in the number and characteristics of children in residential care institutions after an emergency. The design of the data collection approach was adapted from a study to estimate the number and characteristics of children in residential care institutions in Cambodia. The pilot was implemented in southern Haiti in April 2017, six months after Hurricane Matthew devastated the region.

## Methods

### Research questions

The central research question was to determine the feasibility of evaluating movement into residential care following a natural disaster. An appropriate tool would be able to generate representative information about the scale and nature of children entering residential care institutions in a given area. Specific child characteristics of interest included age, sex, parental status, original location, date of arrival, person who placed the child in the facility, and reason for separation. Date of arrival was analyzed to determine when the child had arrived in relationship to the emergency (i.e., before or after).

### Context

The pilot study took place in the Sud Department of Haiti in April 2017. The Sud Department is one of the poorest areas of Haiti. It has a population of approximately 775,000 people. Six months prior to the pilot, Sud was severely affected by Hurricane Matthew, with widespread devastation to property, livestock and livelihoods.

Sud had at least 50 residential care institutions known to the government in 2012, with the true number of institutions at the time of data collection expected to be even greater. Prior to the hurricane, concerns about trafficking and abuse of children in residential care in the area were high. Reform and de-institutionalization efforts were gaining momentum. The effect of the hurricane on children in residential care in Sud was unknown.

Initially, the research team had planned to conduct the pilot throughout all of Sud, but due to project timelines and staffing limitations, the study area was restricted to Les Cayes commune. Les Cayes commune encompasses Les Cayes city, which serves as the capital of Sud. Les Cayes also contains the highest concentration of residential care institutions in Sud.

### Sampling

The research team attempted to conduct a complete census of residential care institutions in Les Cayes commune. Using a two-stage process, the team created a comprehensive list of institutions that met our operational definition (any facility that offers overnight care primarily to children under 18, with at least one salaried staff or volunteer caregiver). First, the team gathered the names, addresses and contact information of the 17 institutions known to and visited by the Institut de Bien-Être Social et Recherche (IBESR) as of November 2016. (IBESR is the social work division within the Government of Haiti.) Second, this official list was supplemented by key informant interviews in the communities to identify additional institutions that may be unknown to IBESR or that may fall outside of the government’s definition of residential care institutions (e.g., transit centers). Informants included shopkeepers, religious leaders, and people who lived in close proximity to institutions reported by other informants. Ten additional institutions were identified through these interviews.

### Data collection and tools

Following the finalization of the list of institutions, the research team trained 10 local data collectors to carry out the study over a two-week period. Per government protocol, a representative from IBESR accompanied the data collectors to all institutions. At each facility, the data collectors attempted to administer four separate tools: a group count tool, a record review tool, interviews with staff, and interviews with children 10 years of age and older (Table 1). Using different tools allowed the researchers to compare and triangulate multiple sources of information. All tools required written informed consent from the institution director before proceeding. The directors serve as the legal guardians of the children who live at their facilities and therefore their consent was a prerequisite for interacting with any children in their institution. In addition, for the interviews with children, each child was asked for individual written assent. Staff members who participated in the staff interviews were also asked for written informed consent.

**Table 1:** Tools for data collection.

Tool	Respondents	Description
Group Count	All children present	A direct count of children in each institution, whereby all children were requested to gather in a central location. Following a brief introduction of the project, the children were asked to raise their hand or otherwise self-identify if they had arrived in the institution after Hurricane Matthew.
Record Review	Child records	A review of any records on the institution premises that included relevant child characteristics, including date of arrival, in order to identify children who had arrived after Hurricane Matthew.
Staff Interview	Staff present	An interview with up to two staff members at each institution to determine the number of children in residence before and after Hurricane Matthew.
Child Interview	All children 10 years of age and older and present	An interview with any child who was at least 10 years old and present at the time of the visit, with questions addressing their particular experience, characteristics and date of arrival.

The project was led by researchers from Columbia University and Save the Children. In Haiti, local partners included Save the Children Haiti, UNICEF and IBESR. The study protocol was approved by the Institutional Review Board at the Columbia University Medical Center (AAAQ9606).

## Results

Due to missing data and other limitations, the results generated by this study do not provide robust information about the scale and nature of children entering residential care in Les Cayes commune following Hurricane Matthew. However, the study did generate significant learning regarding the feasibility of the tools. The results are therefore organized around three elements of feasibility that affected the quality of the data: institutional accessibility, children’s availability and government influence. Comparability of the findings from the four different tools is also discussed.

### Institutional accessibility

Residential care institutions often have concerns about sharing information with outside organizations, including government bodies. Although the presence of official representatives may have reduced this challenge, a substantial number of individual institutions refused to participate in the study (n = 5, 18.5% of the 27 known institutions). As a result, the results should be interpreted in light of potential selection bias towards cooperative institutions. Among the 22 institutions that were willing to participate, only seven institutions (32%) had any records available for the children living in the facility. Reasons given for not having records available included storage offsite or in a locked room, confidentiality concerns and, for at least one institution, destruction of records during Hurricane Matthew. Even within the seven institutions where records were available, the quality of the records was often dubious (e.g., missing fields and contradictory information, such as children whose arrival dates were listed as prior to their date of birth).

### Children’s availability

Two of the tools (the group count and the child interview) required direct child participation. However, due to both security concerns and competing responsibilities for IBESR staff, data collection was restricted from 9am to 2pm each day. These limited working hours overlapped with the school day. The overlap was particularly problematic for the child interviews since it was considered ethically inappropriate to remove children from class for interviews. As a result, in slightly fewer than half of the institutions visited (n = 10 institutions), staff reported that at least some eligible children could not be interviewed. The exact proportion of children who were not interviewed cannot be calculated because there was no master list tallying all eligible children. Although the group count also needed to be conducted outside of class, at most institutions, it was possible to schedule the group count during a short break in the school day, such as lunch, recess or in-between periods. (The group count took approximately 15–30 minutes to complete, depending on the institution’s size.) At the two institutions where the group count was not conducted, this was because the majority of children were participating in offsite activities during data collection.

### Government influence

Over the past several years, the Government of Haiti has taken clear steps to assert its responsibility over the management of residential care institutions. Government responsibility has positive implications for the success of broader de-institutionalization efforts in Haiti and child protection in general and should be commended. At the same time, the government’s prominent role in this study may have compromised some aspects of the original design. For example, the data collection schedule was managed by IBESR representatives and the research team suspected that priority was given to institutions with positive relationships with IBESR. Institutions with a more contentious history with IBESR were visited last, and, in some cases, data collection at these places was rushed. In addition, IBESR representatives called some institutions to notify them of the planned data collection visit at least 24 hours in advance. This notice may have allowed institutions to make alterations to their normal practice, such as developing scripted responses or adjusting the number of children present.

### Comparability across tools

Given the challenges involved in data collection, it is not surprising that the prevalence of new arrivals since Hurricane Matthew varied significantly across the four tools, ranging from 0.69% according to the aggregated child interviews to 20.96% according to the aggregated staff interviews (Table 2). All reports of new arrivals were concentrated in 16 out of the 22 institutions where data were collected, including one institution that opened after Hurricane Matthew. Among these 16 institutions, there were no institutions where the number of new arrivals was consistently reported across all four tools and only four institutions where the number of new arrivals was consistently reported across two tools (group count and staff interviews) (Table 3). However, among the six remaining institutions where no new arrivals were reported, there was agreement across tools about arrivals. Still, there were discrepancies in reports about the current child population at all institutions, including the institutions with no new arrivals.

**Table 2:** Comparison of tools for counting children arriving after the emergency event, all institutions.

Tool	n	Children arriving after hurricane		Current child population		Percentage of children arriving after hurricane	
Group Count	20 institutions	32		762		4.20%	
Record Review	7 institutions	9		440		2.05%	
Child Interview^1^	432 children (at 22 institutions)	3		432		0.69%	
Staff Interview^2^	33 staff	136	183	809	873	16.81%	20.96%
		Lower	Upper	Lower	Upper	Lower	Upper

^1^Child interviews were only conducted with children at least 10 years of age and older. None of the other tools had age restrictions.

^2^When possible, the staff interviews were conducted with multiple staff members per institution, leading to a range of values even at a single facility. For this reason, lower and upper bounds of the aggregated data are both reported.

**Table 3:** Comparison of tools for counting children arriving after the emergency event at a single institution.

Tool	Children arriving after hurricane	Current child population	Percentage of children arriving after hurricane
Group Count	5	62	8.06%
Record Review^1^	missing	missing	---
Child Interview	1	47	2.13%
Staff Interview	2	70	2.86%

^1^Records were stored offsite and not available to the research team.

Because the child interview tool was only applied to children 10 years of age and older, some differences were expected in the prevalence of new arrivals detected by the child interview tool, compared with the other tools (which were not restricted by age). In addition, although the group count was applied to children of all ages, younger children may not have been able to adequately recall or self-identify their arrival date. Also, children who were absent from the group count may have biased the results.

## Discussion

The primary motivation for conducting this study was to determine the feasibility of evaluating movement into residential care following a natural disaster. The results indicate that measurement of new arrivals to residential care institutions was not feasible in Haiti following Hurricane Matthew. Moreover, many of the challenges encountered are likely to be encountered in humanitarian emergencies in other settings. Therefore, the research team does not recommend scale-up of these methods in most humanitarian settings. While there may be limited circumstances in which the tools may be applicable, we caution potential users to carefully consider the factors surrounding residential care in their countries before proceeding with a similar study.

First, the fact that 18.5% of institutions in this sample did not consent to the research is probably not unusual for a humanitarian emergency and may even represent a higher rate of participation than would be expected in other places where government involvement in data collection is not realistic. The issues regarding the availability of records and children are also not unique to Haiti. A recent global review of data on children in formal alternative care found that administrative systems for tracking the number of children in institutions are often very weak and that data quality assurance processes are largely inadequate [17]. Moreover, nighttime travel is restricted in almost all humanitarian settings, so unless children are not in school or weekend working hours are possible, scheduling time with children will be difficult. The success of similar methods in development settings has relied on relatively complete records and greater freedom of movement outside of normal business hours [10].

Second, sampling and representativeness are major concerns for these methods, regardless of the study location. In Haiti, the research team was fortunate that the government had a relatively recent list of institutions in Les Cayes that served as a starting point for data collection. However, even in this situation, it was necessary to supplement the initial government list with institutions provided by key informants. In many settings, government lists will be substantially more incomplete than in Haiti and key informant interviews may not be able to generate adequate coverage of the area of interest. In addition, in large geographic areas, key informant interviews are extremely labor-intensive. Therefore, all other issues aside, in order to use these tools to yield representative data, one would either need to have a fairly comprehensive government list of institutions, or the study area would need to be limited in size.

Third, there were a few issues that may be unique to Haiti. Specifically, residential care in Haiti is a topic that has been under international scrutiny, especially since the discovery of several high-profile child trafficking cases around the time of the 2010 earthquake [18–20]. While this scrutiny has led to some improvements in government oversight of residential care institutions in Haiti, it has also created an environment within institutions of heightened mistrust of outside visitors. In addition, there is a general feeling of assessment fatigue amongst residential care directors in Haiti. One NGO operating in Haiti was worried that conducting this study against the backdrop of these issues would make institution directors less likely to allow other partners to access their institutions in the future, thereby compromising important de-institutionalization efforts in the region.

In light of these concerns, the research team closely reviewed the risk mitigation strategies that were in place to protect children and to ensure thorough and appropriate informed consent. Data collectors were trained to emphasize to directors and staff that this study was not part of a wider quality assessment or investigation of institutions. This was done to mitigate suspicions about the content of the questions asked during the child interviews and thus hopefully mitigate physical abuse of children from directors who were apprehensive about children’s disclosures. Furthermore, all data collectors were also trained on urgent action referrals and direct observation. Data collectors were instructed to immediately report to their team leader any case where a child being interviewed was visibly upset or distressed and to stop the interview.

Although no urgent action referrals or other child protection concerns were identified during this study, these issues underlined some of the particular sensitivities around doing research in residential care institutions in Haiti and elsewhere. Detailed child safeguarding protocols need to be in place in any study in any setting. Specifically, the risks for children associated with the research need to be assessed in relationship to the expected benefits. Finally, unless the robustness of the tools used in this study can be improved, the expected benefits are severely limited because no conclusions can confidently be drawn from the data.

## Conclusion

In conclusion, movement into residential care institutions may increase after emergencies, placing children at risk for abuse, exploitation and other harmful developmental outcomes. However, the extent of this movement is unknown. This was the first known pilot of methods to attempt to measure changes in the number and characteristics of children in residential care following an emergency. While the findings suggested that the tools would not be feasible in most humanitarian settings, the challenges faced did provide a useful source of learning that may inform approaches to research on other topics in residential care institutions. In the event that a humanitarian emergency occurs in a setting with an updated register of residential institutions and high quality child records, and there is an implementing partner (government or otherwise) who has access to local institutions and availability to work outside of school hours, these methods may be appropriate. Historically, most humanitarian emergencies have tended to occur in settings with poor pre-existing governance systems and weak registries and records for residential care institutions, but certainly, there are exceptions to this pattern and the authors would support further piloting of these methods in such scenarios.

Developing representative methods to estimate the magnitude and characteristics of children who enter residential care in humanitarian settings that do not meet these measurement conditions remains an area in need of further innovation and testing. The challenges of creating an institutional sampling frame provide an inherent limitation to any methods that rely on data collected directly from residential care institutions. Alternative approaches that incorporate household survey methods to ascertain movement into residential care based on reports from caregivers may be more realistic in places without institutional sampling frames. The work that has already been done to apply household survey methods to the measurement of separated and unaccompanied children provides a starting point for this critical area of research [15]. In the interim, ongoing programmatic efforts to improve government oversight of residential care institutions are recommended.
